# Determinants of Self-Rated Health Perception in a Sample of a Physically Active Population: PLENUFAR VI Study

**DOI:** 10.3390/ijerph15102104

**Published:** 2018-09-25

**Authors:** Carmen Sayón-Orea, Susana Santiago, Maira Bes-Rastrollo, Miguel A. Martínez-González, Maria R. Pastor, Maria J. Moreno-Aliaga, Josep A. Tur, Aquilino Garcia, J. Alfredo Martínez

**Affiliations:** 1 Department of Preventive Medicine and Public Health, School of Medicine-Clínica Universidad de Navarra, University of Navarra, 31008 Pamplona, Spain; mbes@unav.es (M.B.-R.); mamartinez@unav.es (M.A.M.-G.); 2 Department of Nutrition Food Sciences and Physiology, Center for Nutrition Research, University of Navarra, 31008 Pamplona, Spain; ssantiago@unav.es (S.S.); mjmoreno@unav.es (M.J.M.-A.); jalfmtz@unav.es (J.A.M.); 3 Centro de Investigación Biomedica en Red, Área de Fisiopatología de la Obesidad y la Nutrición (CIBEROBN), 28029 Madrid, Spain; pep.tur@uib.es; 4 Instituto de Investigación Sanitaria de Navarra IdiSNA, Navarra Institute for Health Research, 31008 Pamplona, Spain; 5 Department of Nutrition, Harvard T.H. Chan School of Public Health, Boston, MA 02115, USA; 6 Vocalia Nacional de Alimentación, General Pharmaceutical Council of Spain, 28840 Madrid, Spain; rosariopastor@redfarma.org (M.R.P.); aquilinogarcia@redfarma.org (A.G.); 7 Department of Nutrition and Dietetics, Faculty of Sciences and Arts, Catholic University of Avila, 05005 Avila, Spain; 8 Research Group on Community Nutrition & Oxidative Stress, University of the Balearic Islands, 07122 Palma de Mallorca, Spain; 9 Precision Nutrition Program, Research Institute on Food and Health Sciences IMDEA Food, 28049 Madrid, Spain

**Keywords:** self-rated health, physical activity, diet adequacy, lifestyles, nutritional status

## Abstract

The aim of this study was to investigate determinants of self-rated health (SRH) perception in Spanish adults. This cross-sectional study including data from 11,342 participants from the Spanish PLENUFAR VI study. SRH status was grouped in two categories (‘good’/‘poor’) and the associations of socio-demographic characteristics, lifestyles, diet adequacy and chronic disease with SRH were assessed. After adjusting for relevant confounders, the risk ratios (RR) and (95% confidence intervals) for poor SRH were 1.05 (1.03–1.07) for each hour of increment of sitting, 1.56 (1.30–1.88) for short (≥5 h vs. 7–8 h) sleep duration, 0.63 (0.55–0.72) for vigorous (vs. light) physical activity, 0.61 (0.50–0.74) for adequate (vs. non-adequate) diet. Activities like jogging [RR for each unit of increment in the METs-h/day = 0.87 (0.82–0.92)], gymnastics [0.87 (0.81–0.93)], biking [0.91 (0.85–0.98)], and track and field [0.94 (0.89–0.98)], were associated with better health perception. Normally weight participants with any chronic disease had lower probability to report poor SRH than overweight/obese participants with any chronic disease. Frequent consumption of bread (>2 servings/day) was associated with a lower adjusted mean of health perception scale, while higher consumption of vegetables and fruit or fish were associated with higher values, concerning good SRH. We can conclude that normal-weight participants even suffering a chronic disease had lower probability to report poor health perception than participants with overweight/obesity and a chronic disease especially for hypertension and diabetes. Activities like jogging, gymnastics, biking, and track and field, and a higher consumption of fruits, vegetables and fish, were associated with better health rated perception.

## 1. Introduction

Self-rated health (SRH) perception is a measure in epidemiological research that is increasingly used. SRH is based on one question: “How in general would you rate your health?” The responses are usually on a four- or five-point scale, ranging from poor to excellent [1], which then in epidemiological studies it has been dichotomized into ‘good or excellent’ and ‘fair or poor’ [2].

Furthermore, SRH values involve various aspects concerning people’s general health [1]. When health is rated by each person, several issues could influence the rating such as culture, age, gender, educational level, employment status, place of residence, lifestyle factors (smoking, alcohol consumption, and physical activity) the same as biological, psychological and social dimensions [3]. Thus, it has been proved that SRH is a strong independent predictor of mortality, disease-specific mortality and the incidence of a number of chronic diseases such as diabetes and cardiovascular diseases [1,4]. In Spain, according to the last National Health Survey, 74% of the population, rates their health as good or very good [5]. In this context, obesity has been associated with poor SRH [6]. However, until now, nutritional status has not been analyzed at the same time with the prevalence of the most common chronic diseases and the association with the SRH estimations.

Indeed, it is necessary to determine those factors associated with negative perceived health, analyzing further determinants than those analyzed until now, such as diet quality, different types of physical activity and other lifestyle factors, and also it is important to investigate the relation between overweight/obesity and SRH and whether or not this relation varies when other diseases are considered. Therefore, the aim of this study was to identify new factors associated with poor SRH in a physically active Spanish population.

## 2. Materials and Methods

From April 2017 till June 2017, a total of 4102 community pharmacists conducted a survey of a population-based study that included 11,443 physically active men and women from Spain. All participants were involved in the 6th National Plan of Nutritional Education (PLENUFAR VI). This study was part of a campaign promoted by the Spanish Pharmacists Council, this campaign focused on nutritional education and physical activity. The community pharmacists distributed information about nutrition and healthy lifestyles to those who insured that they take part in at least some physical activity. So only those people who practice some physical activity could took part of the survey. Therefore, volunteers were recruited by community pharmacists, who had contact with women and men who went into the pharmacy. All the participants were specifically asked if they would be willing to take part in the study. After ensuring that participants have understood the information, only those who voluntarily accepted were enrolled and received an informed consent (participants did not get any economic benefit from participating). Health professionals were recruited through the Spanish Pharmacists Council to collect data. All of them received a training session and an “application guide”, a document with basic information about the survey, instructions to formulate every question and a decision tree to interpret the result of the information in each case. Furthermore, a videoconference explaining the study was directly broadcasted to every provincial pharmacist college. Additionally, a website (http://www.portalfarma.com/Profesionales/campanaspf/categorias/Paginas/Plenufar-6.aspx) was available for all pharmacists involved in the study to provide materials that assure harmonization among interviewers. This approach has been successfully applied in previous PLENUFAR surveys among older individuals [7] and women in fertile age [8], and perimenopausal or postmenopausal period [9].

The Institutional Review Board of the University of Navarra approved the study protocol (Ref. 2017.034). This National Plan of Nutritional Education was recognized by the Spanish Ministry of Health and Social Activity as a program of State Health Interest.

### 2.1. The Survey

The principal aim of this program was to collect information regarding adequate dietary habits, including the nutritional status, diet adequacy, SRH, and physical activity.

The community pharmacist administrated the survey, and all the information was collected through a questionnaire specifically designed for this purpose, 49 questions categorized into 3 sections were included in the survey. The first section collected information about socio-demographic variables (sex, age, educational level, smoking status, sleeping hours), anthropometric (directly measured weight and height) and clinical and health related variables (medical diagnosis of chronic diseases, chronic medication intake, SRH, and supplements intake), the second section collected information about diet adequacy [10], and the third section collected information about physical activity [11].

Anthropometric measurements were taken by community pharmacists at pharmacies (current weight and height) and body mass index (BMI) was calculated dividing weight in kilograms by the square of height in meters, according to WHO criteria [12]. We used BMI to classify individuals according to their nutritional status as follows: underweight <18.5 kg/m^2^, normal weight: 18.5 to <25 kg/m^2^, overweight ≥25 to <30 kg/m^2^, and obesity ≥30 kg/m^2^.

### 2.2. Diet Adequacy Assessment

Diet adequacy was assessed by the Diet Quality Screener a previously validated tool, that is used in primary care settings to estimate overall diet quality [10]. Participants had to answer the questions supporting their responses on their usual dietary behaviors over the previous 12 months, reporting their habitual intake of 18 food items grouped in three categories. The first category included usual consumption of bread, vegetables, fruits, milk, rice and pasta, vegetable oils, alcoholic beverages and breakfast cereals. The second group included the consumption of meat, sausages, cheese, pastry, butter, sugar sweetened beverages and fast foods, and the third category included fish, legumes and nuts. For the first category, food frequency consumption was grouped into three possible frequencies (<1/day, 1/day and ≥2/day) for the second category the possible frequencies were <4/week, 4–6/week, and ≥7/week, and for the third category the frequencies of consumption were <2/week, 2–3/week, and ≥4/week. Then, based on the frequency of consumption of each item, diet adequacy score was calculated as follows: in the first group except for alcohol intake, daily intake of 1 serving of food scored 2 points while lower consumption scored 1 point and higher consumption scored 3 points, alcohol intake of 1 serving per day scored 3 points and lower and higher intakes scored 1 point. The second group was considered as detrimental food in a dietary pattern, therefore, the scoring was as follows: less than 4/week scored 3 points, 4–6/week scored 2 points and ≥7/week scored 1 point. The food items included in the third category were considered as beneficial for health, therefore the scoring was as follows: the highest consumption (≥4/week) scored 3 points, while 2–3/week scored 2 points and less than twice per week scored 1 point. All food items scores were summed up and the total possible score ranged from 18 to 54 points, as detailed elsewhere [10].

### 2.3. Physical Activity Assessment

Leisure-time physical activity (PA) was assessed using a previous validated questionnaire [11], including questions to collect information about the type of activity, the duration (hours per week) and the frequency (months per year). Trained interviewers collected the required information about 17 types of activities (hiking, jogging, track and field, outdoor and indoor biking, swimming, tennis, soccer, other team sports, aerobics, mountaineering, gymnastics, gardening, skiing, judo, sailing, or other sports). Time spent in each activity in hours per week was multiplied by the typical energy expenditure, expressed in metabolic equivalent tasks (METs), then summed over all activities to yield a METs-h/week score for each participant as previously published [13].

PA intensity was estimated by the ratio between total METs-h/week and total hours of PA per week obtaining the mean of METs of each person. According to PA intensity, activities were categorized into light PA <3.0 MET, moderate PA 3–<6 MET and vigorous PA ≥6.0 MET.

### 2.4. Chronic Deaseses Assessment

The presence of a chronic disease was determined by the community pharmacist asking the participant if they have been diagnosed with any of the following chronic diseases: diabetes, hypertension, hypercholesterolemia or other.

### 2.5. Outcome Assessment: Self-Rated Health

The following question was used to determine SRH: “In general, how would you describe your health?” The five possible answers were: “excellent”, “very good”, “good”, “fair”, and “poor”. For the analyses where SHR was considered a continuous variable, “poor” was coded as 1, “fair” as 2, “good” as 3, “very good” as 4, and “excellent” as 5. For the poisson regression analyses, we dichotomized these responses into “good perception” (including excellent, very good and good answers vs. “poor perception” (including fair and poor answers).

### 2.6. Data Collection

The questionnaires were available online, through https://formacion.nodofarma.es, where all the pharmacists participating in the survey had a personal password, to enter the data of each participant. A total of 11,443 men and women were included in the study, 101 participants were excluded because of implausible values on weight (*n* = 4), height (*n* = 3), or age being out of the predefined limits <17 years old (*n* = 94), therefore, the final sample for the present analysis was 11,342 participants.

### 2.7. Statistical Analyses

Mean values and standard deviations (SD) for continuous variables and percentages for categorical variables were used as descriptive statistics. T test or χ^2^ tests were used to compare the characteristics of the participants according to their self-rated health perception (two groups) and d Cohen or phi Cramer coefficients were calculated to estimate the effect size difference between the compared groups.

Poisson regression models were conducted to estimate the relationships between sociodemographic variables and SRH. The risk ratios (RRs) and 95% confidence intervals (CIs) for poor health perception were calculated. For all analyses, we fitted a crude univariate model, and a multivariable model after additional adjustment for the following potential confounders: sex, age (continuous), educational level (four categories), BMI (kg/m^2^), smoking status (three categories: current, former, never), chronic diseases [diabetes, hypertension, dyslipidemia, other diseases (yes/no)], chronic treatments (yes/no), food intolerances [gluten, lactose and other (yes/no)], use of supplementation (yes/no), sitting hours (continuous), sleeping hours (four categories), diet adequacy score (three categories), and physical activity intensity (three categories).

When we analyzed the association with poor health perception for each unit of increment in the METs-h/day for each physical activity, we fitted a crude univariate model, and a multivariate model adjusted for all the variables included in the first analysis.

Using ANCOVA models, adjusted means and the 95% CI of SRH according to the frequency of consumption of each food item that was included in the food adequacy score were calculated.

The probability of poor health perception and the nutritional status was also analyzed using the same multivariate model of the previous analyses only the BMI was excluded from the model.

Restricted cubic splines were conducted to calculate the RR and 95% CI for poor health perception and the METs-h/day used as continuous variable. For this analysis we considered those who spent 3 METs-h/day as the reference category. The results were adjusted for the same potential confounders as the main Poisson regression analyses.

For the last analyses, the participants were classified as normal weight and healthy, and compared the probability of poor health perception with those who were normal weight and who also suffered from diabetes, or hypertension or hypercholesterolemia or other diseases. Also, the normal weight and healthy participants were compared with those participants who were overweight or obese and also suffer from diabetes or hypertension or hypercholesterolemia or other diseases. The analyses were adjusted for the same multivariate model of the previous analyses except for the specific disease analyzed at each time. All *p* values presented are two-tailed; *p* < 0.05 was considered statistically significant. Analyses were performed using STATA/SE version 12.0 (StataCorp Inc., College Station, TX, USA).

## 3. Results

The characteristics of the participants according to the health perception status (“good” and “poor” health perception) are shown in Table 1. The mean age of all the participants included in the study was 43.2 (SD: 14.9) years old, 52.8% being women and the mean BMI being 24.6 (SD: 4.0) kg/m^2^. Participants who reported having a poor health perception compared to those who reported having a good health perception were more likely to be women, older, less educated, with a higher BMI, to be current or former smokers, whose prevalence of chronic diseases, and food intolerances were higher, spent more hours sitting and less hours sleeping, had lower diet adequacy score, and were less physically active (Table 1). Meaningful effect size between the groups were only observed in BMI (d Cohen = 0.85).

The RR for poor health perception and sociodemographic, lifestyles and health related factors are shown in Table 2. After adjusting for potential confounders, the RR for poor perception in university graduates in comparison with those who had less than primary studies were 0.67 (95% CI: 0.54–0.82). All chronic diseases were associated with higher probability of poor health perception [diabetes 1.39 (1.18–1.64), hypertension 1.34 (1.15–1.56), dyslipidemia 1.31 (1.14–1.50)]. Interestingly, when sitting hours were analyzed the RR was 1.05 (1.03–1.07) for each additional hour of sitting. Short sleep duration (≤5 h/day) was associated with higher risk of poor health perception 1.56 (1.30–1.88) in comparison to sleeping 7–8 h. An adequate diet (≥44 points of the Diet Adequacy Score) was associated with a 39% lower probability of having poor health perception in comparison with those whose diet was not adequate (<38 points) [RR = 0.61 (0.50–0.74)]. Similarly, moderate and vigorous physical activity was associated with a lower probability of poor health perception in comparison with those who only participate in light physical activity RR = 0.80 (0.70–0.91) and 0.63 (0.55–0.72) respectively. All the variables included in the model explained 19% of the variance in SRH.

In Table 3, the RR for poor health perception for each unit of increment of METs/day of each different type of physical activity are shown. After adjusting for potential confounders, the activities which were associated with a lower probability to report poor health perception were jogging [0.87 (0.82–0.92)], gymnastics [0.87 (0.81–0.93)], biking [0.91 (0.85–0.98)], and track and field [0.94 (0.89–0.98)]. In the other hand judo was associated with higher probability to report poor health perception [1.11 (1.01–1.21)].

**Figure 1:**
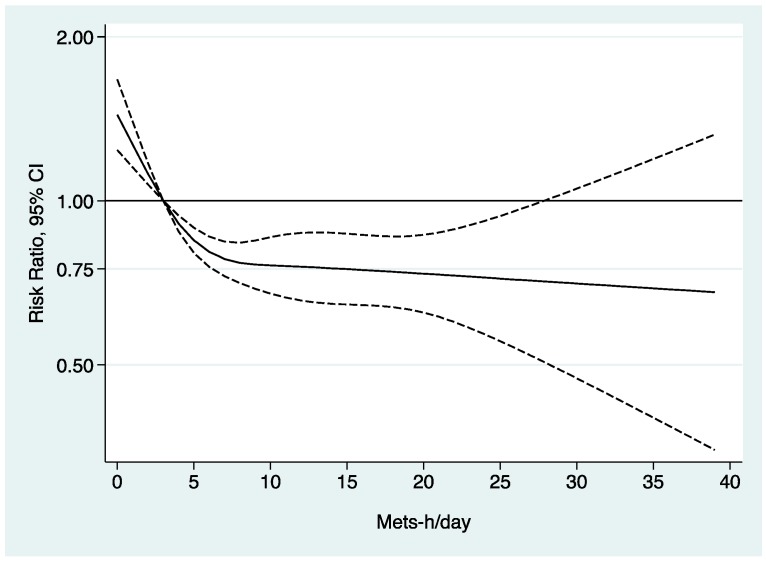
Association between total METs-h/day and the probability of reporting poor SRH. Continuous black line represents the RR and the pointed-lines represent the 95% confidence interval. Adjusted for sex, age, educational level, Body mass index, smoking status, diabetes, hypertension, dyslipidemia, other diseases, gluten, lactose or other intolerances, supplements, chronic treatment, sleeping hours, time spent sitting, healthy diet adequacy score.

When we analyzed total METs-h/day as a continuous variable using a cubic spline model, we found that compared to those who spend 3 METs-h/day those who spend more METs-h/day had lower probability to report poor self-rated health, and those who spend less than 3 METs-h/day had a higher probability of reporting poor self-rated health (Figure 1).

**Figure 2:**
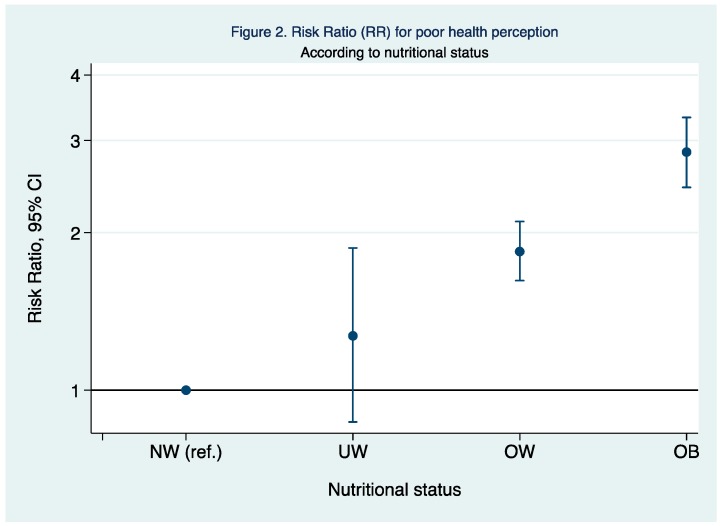
RR and 95% CI for poor health perception according to the nutritional status. NW, Normal weight; UW, Underweight; OW, Overweight; OB, Obese. Adjusted for sex, age, educational level, smoking status, diabetes, hypertension, dyslipidemia, other diseases, gluten, lactose or other intolerances, supplements, chronic treatment, sleeping hours, time spent sitting, physical activity, and healthy diet adequacy score.

When the risk of poor health perception was analyzed according to the nutritional status, those participants being overweight and obese had a 1 to 3 fold higher probability in comparison with those who had normal weight (Figure 2).

**Figure 3:**
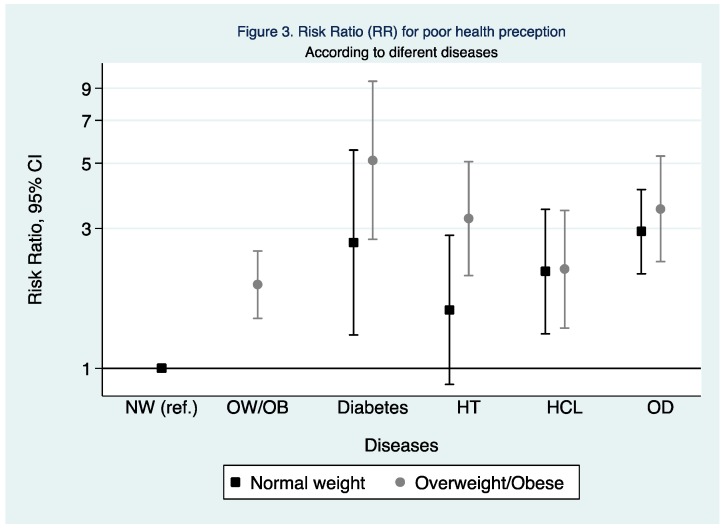
RR and 95% CI for poor health perception comparing healthy and normal weight participants to overweight/obese participants, and to normal weight and overweight/obese participants plus diabetes, hypertension hypercholesterolemia, and other chronic diseases and BMI. NW, Normal weight; OW/OB, Overweight/Obese; HT, Hypertension; HCL, Hypercholesterolemia, OD, Other diseases. Adjusted for sex, age, educational level, smoking status, diabetes (except in the diabetes analysis), hypertension (except in the hypertension analysis), dyslipidemia (except in the hypercholesterolemia analysis), other diseases (except in the hypertension analysis), gluten, lactose or other intolerances, supplements, chronic treatment, sleeping hours, time spent sitting, physical activity, and healthy diet adequacy score.

The joint effect of nutritional status and the prevalence of some chronic diseases were assessed. The normal weight participants without any self-reported chronic disease were compared with the participants who were a normal weight, but had diabetes, hypertension, hypercholesterolemia or other diseases. The same healthy participants with a normal weight were then compared with the participants who were overweight or obese, as well as suffering from diabetes, hypertension, hypercholesterolemia or other diseases. As illustrated in Figure 3, compared to those participants with a normal weight and healthy, the normal weight participants who suffered from diabetes or hypercholesterolemia or other diseases had a 1 to 3-fold higher probability of reporting a poor health perception. When the normal weight and healthy subjects were compared with those in the overweight/obese category and reporting any chronic disease, a 2 to 5 fold higher probability of reporting a poor health perception were observed.

When we calculated the adjusted means of health perception [being 1 ‘poor’ perception and 5 ‘excellent’ health perception] according to the frequency of consumption of some types of food it were found that a higher consumption of bread (≥2/day) was associated with lower mean health perception (*p* for trend <0.001). On the other hand, a higher consumption of vegetables and fruits (≥2/day) was associated with a higher average health perception (*p* for trend <0.001 and 0.002 respectively). Consuming fish 4 or more times a day was also associated with a higher average of health perception (*p* for trend 0.006) The rest of the studied food items did not show any association. However, in all of the food items studied, the magnitude of the effect was very small (data not shown).

## 4. Discussion

The results from this cross-sectional study revealed that SRH is associated with diverse factors, some of them studied before and some of them newly described here, such as different types of physical activity, several food items, and the occurrence of chronic diseases at the same time with unhealthy nutritional status.

Previously, it was described that physical activity contributes significantly to the self-assessment of health. For example Piko [14] found a positive correlation between physical activity and self-perceived health (*r* = 0.20). Similarly, Darviri et al. [15] found that after adjusting for sociodemographic and health related factors, regular exercise was associated with better health ratings. Those who exercise regularly were four times more likely to report an excellent or very good self-rated health than those who do not exercise regularly [15]. Another study found that those who did not exercise >1 h/week had 2 times more probability to report a poor SHR than those who exercised 1 or more hours per week [16]. In the same way, another study conducted in Sweden found nearly the same association, those who were physically inactive defined as those who practiced <2 h/week in comparison with those who practiced a vigorous exercise had higher odds to report a poor SRH (OR = 2.8 (95% CI: 2.3–3.3) [17]. Our findings are in the same line of previous studies, finding that moderate and vigorous physical activity intensity was associated with 20% and 37% lower probability to report a poor SRH, respectively than those who practiced light intensity physical activity. However, in addition to what is already known, the results presented found that the physical activities which were more associated with a better SRH were jogging and gymnastics followed by biking and track and field. In this context, it was found that self-reported health in those who practice outdoor physical activities was not significantly greater than those who practice indoor physical activities [18], these results are in accordance with our findings because the type of physical activities that we found were more associated with a better self-rated health were both outdoor (jogging, biking, and track and field) and indoor activities (gymnastics). When we conducted the cubic splines analysis, our results confirmed the actual recommendations from WHO (150 min/per week of a moderate intensity activity in adults) [19], with our results we observed that those who practice ≥3 METs-h/day of any physical activity had less probability to report poor self-rated health in comparison with those who spend less than 3 METs-h/day. This might be translated to practice 30 min per day of a moderate intensity activity (i.e., walking 6 km/h).

Regarding diet adequacy, it was found that a healthy dietary pattern was associated with a higher probability of reporting very good SRH [15], with the same trend our results found that a high diet adequacy score was associated with 39% lower odds of reporting a poor health perception in comparison to those with a low diet adequacy. When each food item in relation to SRH was studied, vegetable, fruits and fish consumption was associated with a higher mean health perception score, and, on the contrary, higher consumption of bread was associated with a lower mean of the health perception score. One study that specifically assessed the consumption of fruits and vegetables and SRH found that each additional daily serving of fruit and vegetables was associated with a higher odds of reporting health as good or better in both women and men, by 9% and 10% respectively [20].

Some of the lifestyle factors that we studied showed that those participants who had very short sleep duration (≤5 h/day) had a 1.6 times higher probability to report a poor health perception than those who sleep 7–8 h/day. In line with our results several studies have found that sleep dissatisfaction is strongly related with poor SRH [15,16]. Also, we identified that those who spent more time sitting were more prone to report a worse SRH. However a previous study did not find an apparent association between this. The authors of that study did not expect that result, especially because growing evidence of an association between sedentary behavior and different health outcomes exists [21].

Our study revealed that nutritional status was strongly associated with SRH. This association was previously observed in several investigations [6,15,16,17,20]. Also, Stefan et al. [22] featured inverse association between many body composition parameters such as weight, BMI, and fat mass percentage with SRH.

An important finding of our study appeared when the joint association between nutritional status and some chronic diseases were analyzed. In comparison to those who were healthy and of a normal weight, having any chronic disease among normal weight participants was associated with a higher probability to report poor SRH, except for hypertension, but notably, when a chronic disease was added to being overweight or obese the probability of reporting a poor SRH was increased, especially in the case of diabetic and hypertensive participants. The association between healthy and unhealthy participants and SRH was previously explored by several studies [15,16,20,23], suggesting that the presence of one or more comorbidities was related to a higher probability of reporting poor SRH.

### Strengths and Limitations

A limitation of our study is the cross-sectional design; therefore, it is not possible to conclude whether the factors that we studied were causes or consequences of poor self-rated perception. Another possible limitation is the large number of examiners that might cause an inter-observer variation, however, each examiner received a complete guide of the questionnaire items and a training session explaining how to administer the survey to avoid possible bias. The origin of the recruitment of the volunteers (pharmacies) might result in a non-representative sample of physically active population, therefore, the generalizability of our results should be interpreted with caution Compared to the Spanish population, the included sample had a lower prevalence of chronic diseases, and had a lower BMI mean [5]. The strengths of the present study are the large number of participants included in the analysis, the objective measurements of weight and height that avoided under- or over-reporting of weight and height data, and the novel approach in investigating chronic diseases and the association with SRH. Furthermore, the adequacy to the diet and physical activity were recorded using validated tools [10,11].

## 5. Conclusions

Based on the results of this study, we can conclude that besides the largely studied lifestyle factors associated with poor SRH such as physical activity, diet, sleep duration and nutritional status, the activities which were associated with better health perception were jogging, gymnastics, biking, and track and field in comparison with the other physical activities. Also, we can conclude that normal-weight participants even with a chronic disease had lower probability of poor health perception than their counterparts with overweight/obesity and also a chronic disease. This was especially found for hypertension and diabetes compared to participants diagnosed with hypercholesterolemia or other chronic diseases. Finally, higher consumption of fruits, vegetables and fish were associated with better health rated perception, whereas white bread was associated with poorer self-rated health perception.

## Figures and Tables

**Table 1:** Sociodemographic characteristics and clinical conditions of the 11,342 participants, according to health perception status: PLENUFAR VI study.

Characteristics and Clinical Conditions	Poor Health Perception (1–2)	Good Health Perception (3–5)	Effect Size ^‡^	*p*-Value
*n*	1484	9858		
Sex (% men)	40.4	48.3	0.05	<0.001
Age (years)	51.9 (16.4)	41.9 (14.2)	0.31	<0.001
Education level (%):			0.13	<0.001
Less than primary	11.0	1.9		
Primary	23.7	11.0		
Secondary	28.0	30.7		
University	37.3	56.4		
BMI (kg/m^2^)	27.4 (4.0)	24.2 (3.5)	0.85	<0.001
Smoking status (%):			0.05	<0.001
Current	20.2	15.3		
Former	27.7	23.5		
Never	52.1	61.2		
Chronic diseases (%):				<0.001
Diabetes	15.5	2.9	0.20	
Hypertension	33.6	9.3	0.25	
Dyslipidemia	22.0	8.1	0.16	
Other diseases	24.2	11.0	0.13	
Gluten intolerance (yes %)	2.2	1.4	0.02	0.019
Lactose intolerance (yes %)	6.0	3.8	0.04	<0.001
Other intolerance (yes %)	6.2	4.4	0.03	0.002
Supplements (yes %)	39.0	50.5	0.08	<0.001
Chronic treatment (yes %)	62.9	24.0	0.29	<0.001
Sitting hours	5.9 (2.9)	5.1 (2.5)	0.30	<0.001
Sleeping hours	7.0 (1.3)	7.2 (1.0)	0.17	<0.001
Healthy Diet Adequacy Score *	38.9 (3.8)	40.1 (3.5)	0.33	<0.001
Physical activity (METs/day)	6.0 (6.3)	8.9 (7.1)	0.43	<0.001

Values are presented as mean (SD), unless otherwise stated. Abbreviations: BMI, Body mass index; METs, Metabolic equivalent of task. ^‡^ d Cohen for continuous variables or Cramér’s phi for categorical variables. * Healthy Diet Adequacy Score ranged from 18 to 54.

**Table 2:** Risk Ratio (RR) and 95% CI of poor health perception associated to socio-demographic and health related factors.

Factors	Crude RR (95% CI)	*p*-Value	Adjusted RR (95% CI) ^†^	*p*-Value
Sex ^‡^	1.32 (1.19–1.47)	<0.001	1.36 (1.22–1.52)	<0.001
Age (years)	1.04 (1.03–1.04)	<0.001	1.00 (1.00–1.01)	0.035
Education level				
Less than primary	Ref.		Ref.	
Primary	0.53(0.44–0.64)	<0.001	0.88 (0.72–1.07)	0.187
Secondary	0.26 (0.22–0.31)	<0.001	0.75 (0.61–0.92)	0.007
University	0.20 (0.16–0.23)	<0.001	0.67 (0.54–0.82)	<0.001
BMI (kg/m^2^)	1.13 (1.12–1.13)	<0.001	1.07 (1.05–1.08)	<0.001
Smoking status				
Current	Ref.		Ref.	
Former	0.90 (0.78–1.05)	0.198	0.81 (0.70–0.95)	<0.001
Never	0.68 (0.60–0.78)	<0.001	0.75 (0.65–0.86)	0.008
Chronic diseases				
Diabetes *	3.88(3.37–4.46)	<0.001	1.39 (1.18–1.64)	<0.001
Hypertension *	3.54 (3.18–3.94)	<0.001	1.34 (1.15–1.56)	<0.001
Dyslipidemia *	2.56 (2.26–2.89)	<0.001	1.31 (1.14–1.50)	<0.001
Other diseases *	2.18(1.93–2.46)	<0.001	1.77 (1.52–2.05)	<0.001
Gluten intolerance *	1.47 (1.04–2.09)	0.030	1.49 (1.03–2.14)	0.033
Lactose intolerance *	1.51 (1.22–1.87)	<0.001	1.53 (1.22–1.92)	<0.001
Other intolerance *	1.36(1.10–1.68)	0.004	1.04 (0.83–1.29)	0.756
Supplements *	0.66 (0.60–0.74)	<0.001	0.92 (0.83–1.03)	0.162
Chronic treatment *	4.14 (3.72–4.60)	<0.001	1.67 (1.42–1.95)	<0.001
Sitting hours	1.10 (1.08–1.12)	<0.001	1.05 (1.03–1.07)	<0.001
Sleeping hours				
7–8 h	Ref.		Ref.	
≤5 h	3.05 (2.55–2.65)	<0.001	1.56 (1.30–1.88)	<0.001
>5–<7 h	1.67 (1.48–1.90)	<0.001	1.29 (1.14–1.46)	<0.001
>8 h	1.87 (1.57–2.21)	<0.001	1.22 (1.02–1.45)	0.0.26
Diet Adequacy Score				
Not adequate (<38 points)	Ref.		Ref.	
Moderately adequate (38–43 points)	0.68 (0.61–0.76)	<0.001	0.74 (0.69–0.90)	<0.001
Adequate (≥44 points)	0.49 (0.40–0.59)	<0.001	0.61 (0.50–0.74)	<0.001
Physical activity intensity				
Light (<3 METs)	Ref.		Ref.	
Moderate (3–<6 METs)	0.57 (0.50–0.65)	<0.001	0.80 (0.70–0.91)	<0.001
Vigorous (>=6 METs)	0.36 (0.32–0.41)	<0.001	0.63 (0.55–0.72)	<0.001

^†^ The adjusted model includes all the variables shown in the table. ^‡^ Men are the reference category. * No is the reference category.

**Table 3:** Risk ratio (RR) and 95% CI for poor health perception of each unit of increase in METs/day of all the physical activities included in the questionnaire.

Physical Activity	Crude RR (95% CI)	*p*-Value	Adjusted RR (95% CI) ^‡^	*p*-Value
Hiking	1.00 (0.96–1.04)	0.949	0.98 (0.94–1.02)	0.381
Jogging	0.72 (0.69–0.76)	<0.001	0.87 (0.82–0.92)	<0.001
Track and field	0.82 (0.78–0.86)	<0.001	0.94 (0.89–0.98)	0.004
Biking	0.74 (0.69–0.80)	<0.001	0.91 (0.85–0.98)	0.013
Indoor Biking	0.87 (0.81–0.93)	<0.001	1.00 (0.93–1.07)	0.944
Swimming	0.75(0.67–0.83)	<0.001	0.92 (0.84–1.01)	0.083
Tennis	0.81 (0.75–0.87)	<0.001	0.94 (0.87–1.01)	0.129
Soccer	0.78 (0.71–0.85)	<0.001	0.97 (0.87–1.08)	0.572
Other team sports	0.80 (0.69–0.92)	0.001	1.02 (0.86–1.22)	0.823
Aerobics	0.80 (0.72–0.89)	<0.001	0.94 (0.84–1.05)	0.281
Mountaineering	0.84 (0.78–0.90)	<0.001	0.96 (0.90–1.02)	0.229
Gymnastics	0.77 (0.72–0.82)	<0.001	0.87 (0.81–0.93)	<0.001
Gardening	1.09 (1.01–1.17)	0.013	1.06 (0.98–1.14)	0.141
Skiing	0.58 (0.47–0.72)	<0.001	0.87 (0.70–1.09)	0.228
Judo	0.94 (0.88–1.01)	0.092	1.08 (1.01–1.1)	0.023
Other sports	0.87 (0.81–0.94)	<0.001	0.98 (0.91–1.05)	0.594

^‡^ Additionally adjusted for sex, age, educational level, Body mass index, smoking status, diabetes, hypertension, dyslipidemia, other diseases, gluten, lactose or other intolerances, supplements, chronic treatment, sleeping hours, time spent sitting, healthy diet adequacy score.
